# Comprehensive bioinformation analysis of homeodomain-leucine zipper gene family and expression pattern of HD-Zip I under abiotic stress in *Salix suchowensis*

**DOI:** 10.1186/s12864-024-10067-x

**Published:** 2024-02-15

**Authors:** Yujiao Wang, Hongjuan Wang, Chun Yu, Xiaoming Yan, Jiasong Chu, Benli Jiang, Jiabao Zhu

**Affiliations:** Department of Cotton Research Institute, Anhui Academy of Agricultural Sciences, 230001 Hefei, China

**Keywords:** *Salix suchowensis*, *HD-Zip* gene, Comprehensive analysis, Abiotic stress, Expression pattern

## Abstract

**Background:**

Homeodomain-leucine zipper (HD-Zip) transcription factors are plant-specific and play important roles in plant defense against environmental stresses. Identification and functional studies have been carried out in model plants such as rice, *Arabidopsis thaliana*, and poplar, but comprehensive analysis on the HD-Zip family of *Salix suchowensis* have not been reported.

**Results:**

A total of 55 *HD-Zip* genes were identified in the willow genome, unevenly distributed on 18 chromosomes except for chromosome 19. And segmental duplication events containing *SsHD-Zip* were detected on all chromosomes except chromosomes 13 and 19. The *SsHD-Zip* were classified into 4 subfamilies subfamilies (I-IV) according to the evolutionary analysis, and members of each subfamily shared similar domain structure and gene structure. The combination of GO annotation and promoter analysis showed that *SsHD-Zip* genes responded to multiple abiotic stresses. Furthermore, the results of qPCR analysis showed that the *SsHD-Zip* I gene exhibited different degrees of expression under salt stress, PEG treatment and heat treatment. Moreover, there was a synergistic effect between *SsHD-Zip* I genes under stress conditions based on coregulatory networks analysis.

**Conclusions:**

In this study, HD-Zip transcription factors were systematically identified and analyzed at the whole genome level. These results preliminarily clarified the structural characteristics and related functions of willow HD-Zip family members, and it was found that *SsHox34*, *SsHox36* and *SsHox51* genes were significantly involved in the response to various stresses. Together, these findings laid the foundation for further research on the resistance functions of willow *HD-Zip* genes.

**Supplementary Information:**

The online version contains supplementary material available at 10.1186/s12864-024-10067-x.

## Introduction

The homeodomain leucine zipper (HD-Zip) transcription factor is one of the larger families of transcription factors in plants, and it contains the highly conserved homeodomain (HD) and leucine zipper (LZ) [1]. The HD consists of 60 conserved amino acids and binds specifically to DNA, and the LZ is a dimeric motif that is critical for the recognition of DNA binding sites [2]. Based on structure and function, the *HD-Zip* gene family can be divided into four distinct subfamilies, namely HD-Zip I to HD-Zip IV. The HD-Zip I subfamily has the simplest gene structure, containing only the HD and LZ domains, and HD-Zip II contains an additional conserved region at the N-terminus [3]. In addition to the HD and LZ domains, HD-Zip III and IV have a START domain associated with sterol binding. However, a C-terminal MEKHLA domain has also been found in the HD-Zip III subfamily [4, 5].

The four subfamily members differ in their respective functions because of the differences in their structure. The *HD-Zip I* gene plays an important role in helping plants adapt to different environmental conditions, including drought, salt stress and temperature extremes [6, 7]. For example, *Zmhdz10* and *Oshox22*, members of the HD-Zip I subfamily in maize and rice, regulated drought and salt tolerance stress in plants through ABA-dependent signaling pathways [8, 9]. *TaHDZ5-6 A*, a member of the HD-Zip I subfamily in wheat, increased drought tolerance when overexpressed in Arabidopsis [10]. Under salt stress, overexpression of *MdHB7-like* (an apple *HD-Zip I* gene) increased photosynthetic efficiency and reduced ROS and Na^+^ accumulation [11]. The results of RNA-Seq and RT-qPCR showed that radish *HDZ17* from HD-Zip I was highly expressed under high temperature and salt stress. Further studies showed that overexpression of *RsHDZ17* improved the heat tolerance of transgenic Arabidopsis [12]. Members of HD-ZIP II subfamily mainly play an important roles in light avoidance or light signalling and plant development [13, 14]. Five of the ten genes of the subfamily in Arabidopsis were response to changes in light quality [15]. The expression of *ATHB2* and *HAT4* was negatively correlated with the R/FR (red light/far-red light) ratio. Moreover, *HAT1* and *ATHB4* had been implicated in the regulation of the shade avoidance response of plants [16, 17]. And *ATHB2* inhibits the germination of seeds of Arabidopsis [18]. It has been reported that the HD-Zip III subfamily is mainly involved in meristem formation, vascular bundle development and leaf polarity development [19]. In soybean, both GmREV-L-1 and GmHB14-L-2 were highly expressed in vascular cambium cells and maintained consistent high expression levels throughout all xylem maturation stages. GmREV-L-1 and GmHB14-L-2 are key components in xylem differentiation [20]. HD-Zip IV subfamily genes had been shown to be specifically expressed in epidermal cells and play an important role in epidermal cell development and trichosome formation [21, 22]. For instance, *SlHDZIV8*, a member of the HD-Zip IV subfamily in tomato, controlled the morphology of multicellular trichomes by regulating the expression of Hairless-2 [23].

Genome-wide investigations of the *HD-Zip* gene family have been carried out in several species, including Arabidopsis [19], *Dendrobium officinale* [24], Ginseng [25], and peach [26]. However, such work has not been carried out in willow. *Salix suchowensis* is a woody plant of the genus Salix, its branches are strong and can be used to weave wickerwork, baskets and agricultural tools, but it can also be used as a windbreak, sand fixing tree species with high ecological and economic value [27, 28]. The completion of whole genome sequencing allowed us to identify and analyse the expression of abiotic stress genes at the whole genome level [29]. However, little is known about the expression, structure and function of the willow *HD-Zip* genes.

In this study, 55 *HD-Zip* genes were identified in the willow genome, and their evolutionary relationships, conserved domains, gene replication, and cis-acting elements were comprehensively analyzed. At the same time, we also investigated the expression patterns of the *HD-Zip I* gene in willow under salt, PEG and heat stress. These results provide a basis for further studies on the expression and tolerance function of the *HD-Zip* genes in willow.

## Results

### A total of 55 *HD-Zip* genes were identified in *S. suchowensis*

A total of 55 *HD-Zip* genes were identified in S. suchowensis by whole-genome retrieval and validation. All putative *HD-Zip* genes contained a homeobox domain (PF00046). The 55 *HD-Zip* genes were named from *SsHox1* to *SsHox55* based on their physical location and conserved domain. The length of these proteins ranged from 153 ~ 877 amino acids (aa), with the coding region sequences corresponding 462 bp ~ 2643 bp. Moreover, the theoretical isoelectric point (pI) of the *SsHox* genes varied from 4.56 to 9.49. Table 1 provided additional information on the characterizatio of *HD-Zip* genes. Additionally, according to the predicted results of subcellular localization, the majority of the willow HD-Zip proteins were located in the nucleus, SsHox26 and SsHox49 were located in chloroplasts, and SsHox29 was located in the cytoplasm (Table S1).

**Table 1:** Details of the identified *HD-Zip* genes in *S. suchowensis*

Name	Gene Identifier	Location	ORF length (bp)	Protein			
				Length (a.a.)	PI	Mol.Wt. (Da)	Exons
SsHox1	willow_GLEAN_10002342	chr13:5209149.5210205	954	317	5.05	36086.77	2
SsHox2	willow_GLEAN_10003886	chr05:2501516.2504989	1428	475	6.56	53823.25	4
SsHox3	willow_GLEAN_10004942	chr17:487110.487631	522	173	6.29	19451.87	1
SsHox4	willow_GLEAN_10005026	chr05:7848777.7849826	789	262	5.71	30538.8	3
SsHox5	willow_GLEAN_10005056	chr11:7473266.7473769	504	167	9.37	19337.64	1
SsHox6	willow_GLEAN_10005194	chr12:274045.274900	774	257	5.32	29284.43	2
SsHox7	willow_GLEAN_10005705	chr06:6228916.6230045	654	217	8.85	25318.29	3
SsHox8	willow_GLEAN_10005832	chr01:12229293.12230670	963	320	8.69	35689.61	4
SsHox9	willow_GLEAN_10006690	chr07:7817079.7817615	537	178	9.17	20350.79	1
SsHox10	willow_GLEAN_10007184	chr18:2092915.2099179	2571	856	5.85	94346.81	18
SsHox11	willow_GLEAN_10007942	chr18:7540908.7545445	948	315	6.95	35491.58	6
SsHox12	willow_GLEAN_10008337	chr17:8082883.8084041	462	153	7.74	17504.55	3
SsHox13	willow_GLEAN_10008909	chr10:5275904.5277549	870	289	6.01	32,954	3
SsHox14	willow_GLEAN_10009247	chr02:8801327.8803781	885	294	9.08	33006.88	4
SsHox15	willow_GLEAN_10009523	chr12:3864207.3865121	795	264	4.56	30333.3	2
SsHox16	willow_GLEAN_10009658	chr16:11668377.11673053	2274	757	5.96	84363.34	11
SsHox17	willow_GLEAN_10009743	chr15:8411038.8415237	2223	740	5.54	81513.69	8
SsHox18	willow_GLEAN_10009808	chr15:1853449.1856582	2127	708	6.53	77753.34	10
SsHox19	willow_GLEAN_10010028	chr04:720621.724207	2040	679	5.51	74826.89	10
SsHox20	willow_GLEAN_10010090	chr05:5707352.5709223	726	241	9.25	27999.9	4
SsHox21	willow_GLEAN_10010591	chr08:7569496.7570759	708	235	5.91	26951.08	2
SsHox22	willow_GLEAN_10011112	chr15:3371825.3373099	930	309	4.83	34765.26	3
SsHox23	willow_GLEAN_10011227	chr11:5749314.5755253	2529	842	5.86	92313.32	18
SsHox24	willow_GLEAN_10011792	chr02:9983842.9990080	2490	829	5.94	90567.88	10
SsHox25	willow_GLEAN_10012615	chr07:440589.441742	846	281	8.57	31623.73	3
SsHox26	willow_GLEAN_10013067	chr04:11864612.11872078	2613	870	5.91	95497.31	18
SsHox27	willow_GLEAN_10013903	chr07:5920647.5922183	1014	337	4.83	37882.69	3
SsHox28	willow_GLEAN_10014069	chr01:4791570.4792971	1038	345	8.69	38266.97	4
SsHox29	willow_GLEAN_10014688	chr16:11311833.11318838	2556	851	6.06	93492.87	18
SsHox30	willow_GLEAN_10014867	chr16:7124426.7129402	2412	803	5.52	89332.98	11
SsHox31	willow_GLEAN_10015140	chr17:4534451.4535861	732	243	5.91	27663.95	2
SsHox32	willow_GLEAN_10015458	chr08:6462504.6465411	699	232	8.54	25539.89	4
SsHox33	willow_GLEAN_10016083	chr14:3617075.3622033	2499	832	5.76	90238.59	9
SsHox34	willow_GLEAN_10016271	chr14:5066221.5067038	702	233	5.26	26708.69	2
SsHox35	willow_GLEAN_10016696	chr14:1979359.1983173	1017	338	8.38	37672.41	5
SsHox36	willow_GLEAN_10016966	chr02:11557986.11558804	711	236	4.88	27134.03	2
SsHox37	willow_GLEAN_10017080	chr01:20984214.20990008	2634	877	6.03	96673.33	17
SsHox38	willow_GLEAN_10017524	chr01:22569925.22570446	522	173	8.42	20055.49	1
SsHox39	willow_GLEAN_10017797	chr16:8705805.8707465	906	301	8.84	33448.78	4
SsHox40	willow_GLEAN_10019718	chr12:1153935.1157059	2127	708	6.19	77667.02	10
SsHox41	willow_GLEAN_10020038	chr02:14549645.14553648	2283	760	5.49	83273.13	11
SsHox42	willow_GLEAN_10020281	chr14:7939892.7944022	2280	759	5.57	82998.92	11
SsHox43	willow_GLEAN_10020955	chr02:6588033.6589139	939	312	6.28	35742.99	3
SsHox44	willow_GLEAN_10021056	chr02:7575862.7576910	807	268	8.02	30076.02	3
SsHox45	willow_GLEAN_10021385	chr10:6571681.6573779	678	225	8.28	25419.91	4
SsHox46	willow_GLEAN_10021964	chr03:2109069.2113988	1998	665	6.88	73596.48	10
SsHox47	willow_GLEAN_10021975	chr03:2292482.2298568	2418	805	6.18	88596.3	17
SsHox48	willow_GLEAN_10022161	chr03:4404415.4406099	909	302	8.2	33782.95	4
SsHox49	willow_GLEAN_10022766	chr09:1442600.1449942	2688	895	5.97	98426.55	18
SsHox50	willow_GLEAN_10022837	chr09:2382283.2383617	1047	348	6.33	38307.18	4
SsHox51	willow_GLEAN_10023610	chr16:3863371.3864068	582	193	9.26	22556.03	2
SsHox52	willow_GLEAN_10025698	chr03:5546441.5550860	2427	808	5.53	89816.92	11
SsHox53	willow_GLEAN_10025934	chr06:11760197.11761563	1020	339	9.49	37438.11	4
SsHox54	willow_GLEAN_10026270	chr06:14407519.14413810	2436	811	5.8	89369.02	18
SsHox55	willow_GLEAN_10026836	chr06:3548298.3549461	648	215	8.79	24778.76	3

### Phylogenetic analysis and chromosomal distribution of the *HD-Zip* gene family

**Fig. 1:**
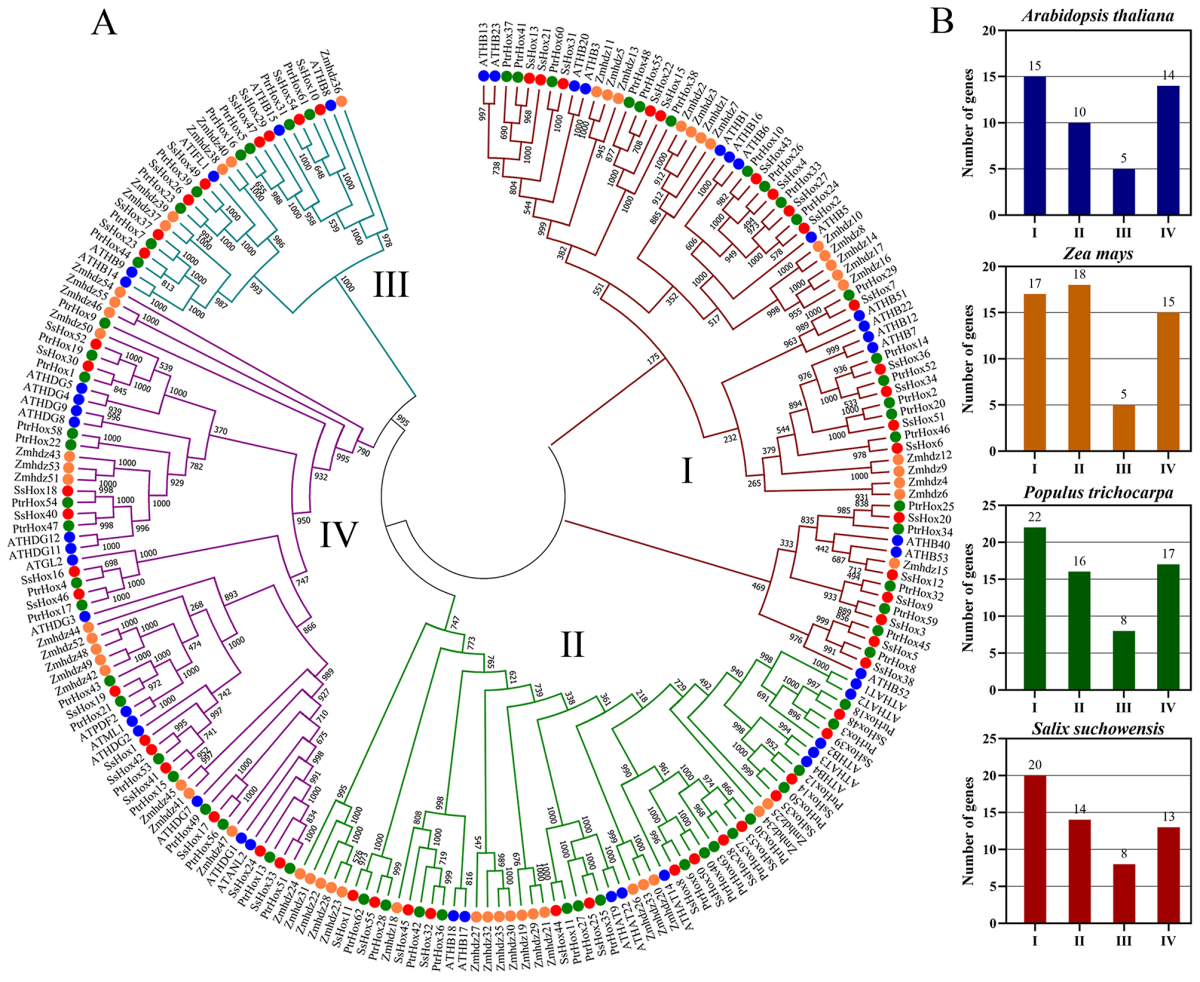
Phylogenetic tree of *HD-Zip* genes from willow, Arabidopsis, poplar and maize. (**A**) Classification of HD-Zip gene family based on phylogenetic tree. 55 *SsHD-Zip* genes, 44 *AtHD-Zip* genes, 63 *PtHD-Zip* genes and 55 *ZmHD-Zip* genes are clustered into four subfamilies (I-IV). *HD-Zip* genes from *S. suchowensis*, Arabidopsis, poplar and maize are denoted by red, blue, green and yellow shape, respectively. The tree was generated with the Clustal X 2.0 software using the neighbor-joining (N-J) method. (**B**) The number distribution of *HD-Zip* gene family in four species

To further understand the evolutionary relationships among HD-Zip family members, a phylogenetic tree was constructed using the 44 Arabidopsis HD-Zip proteins, 55 maize HD-Zip proteins, 63 poplar HD-Zip proteins and 55 willow HD-Zip proteins identified in this study. Using MEGA 11.0, the phylogenetic tree was built using the Maximum Likelihood (ML) and Neighbor-Joining (NJ) methods, respectively. With the exception of a few modest adjustments at internal branches, the tree topologies generated by the two algorithms were essentially similar. The classification results were the same for both methods, the NJ phylogenetic tree is shown in Fig. 1, and the ML phylogenetic tree is shown in Fig. S1. The result showed that the willow *HD-Zip* gene family can be divided into four subfamilies (I-IV), each containing 20, 14, 8, and 13 members of the willow *HD-Zip* gene family, respectively (Fig. 1A). According to the classification of maize, Arabidopsis and poplar, subfamily I was further subdivided into eight clades, designated α, β1, β2, γ, δ, ε, ζ and φ. And the clade β1 and ζ did not contain any willow or poplar *HD-Zip* genes (Fig. S2). The results were consistent with those of Arabidopsis and poplar, with subfamily I among them being the largest and subfamily III the smallest (Fig. 1B). However, the *HD-Zip* gene members in maize were the largest in the subfamily II. In addition, the phylogenetic tree showed that *PtHox* and *SsHox* genes were evenly clustered together, indicating that poplar and willow are closely related.

**Fig. 2:**
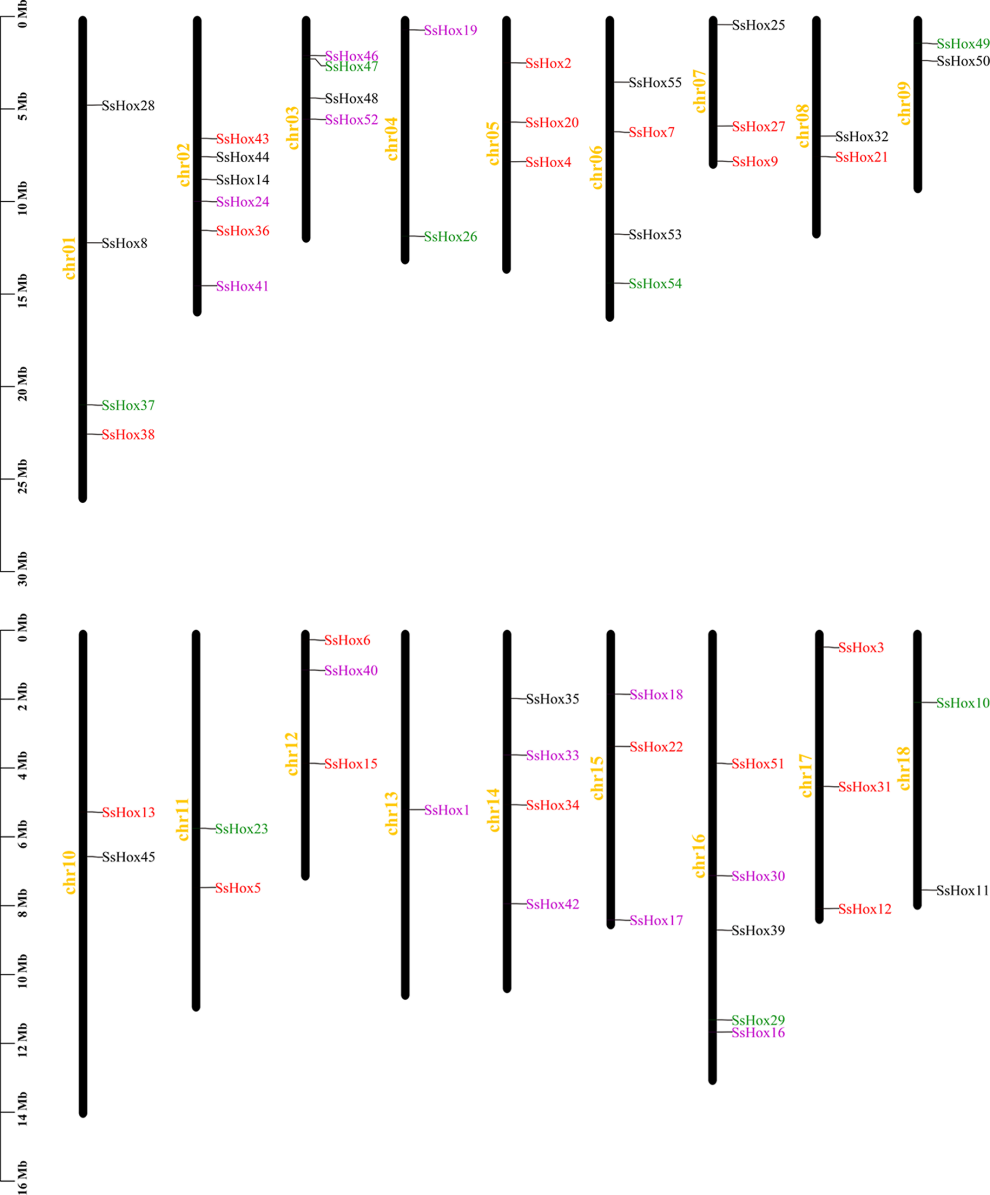
Chromosomal location of *HD-Zip* genes in willow. The 55 *SsHD-Zip* genes are widely mapped to 18 chromosomes of *S. suchowensis*. Different colors represent different subfamilies

According to the *SsHoxs* genome annotation file obtained in the willow database, the locations of the 55 willow *HD-Zips* on the chromosomes were mapped (Fig. 2). The result demonstrated that, with the exception of chromosome 19, the 55 *SsHox* genes were randomly and unevenly distributed on 18 of the 19 chromosomes. Furthermore, Chr02 contains the maximum number of *SsHox* genes with a total of six, while the chr13 had only one gene. Specifically, both chr05 and chr17 had three *HD-Zip* genes, all belonging to subfamily I.

### Conserved motifs, domains, and gene structural analysis

**Fig. 3:**
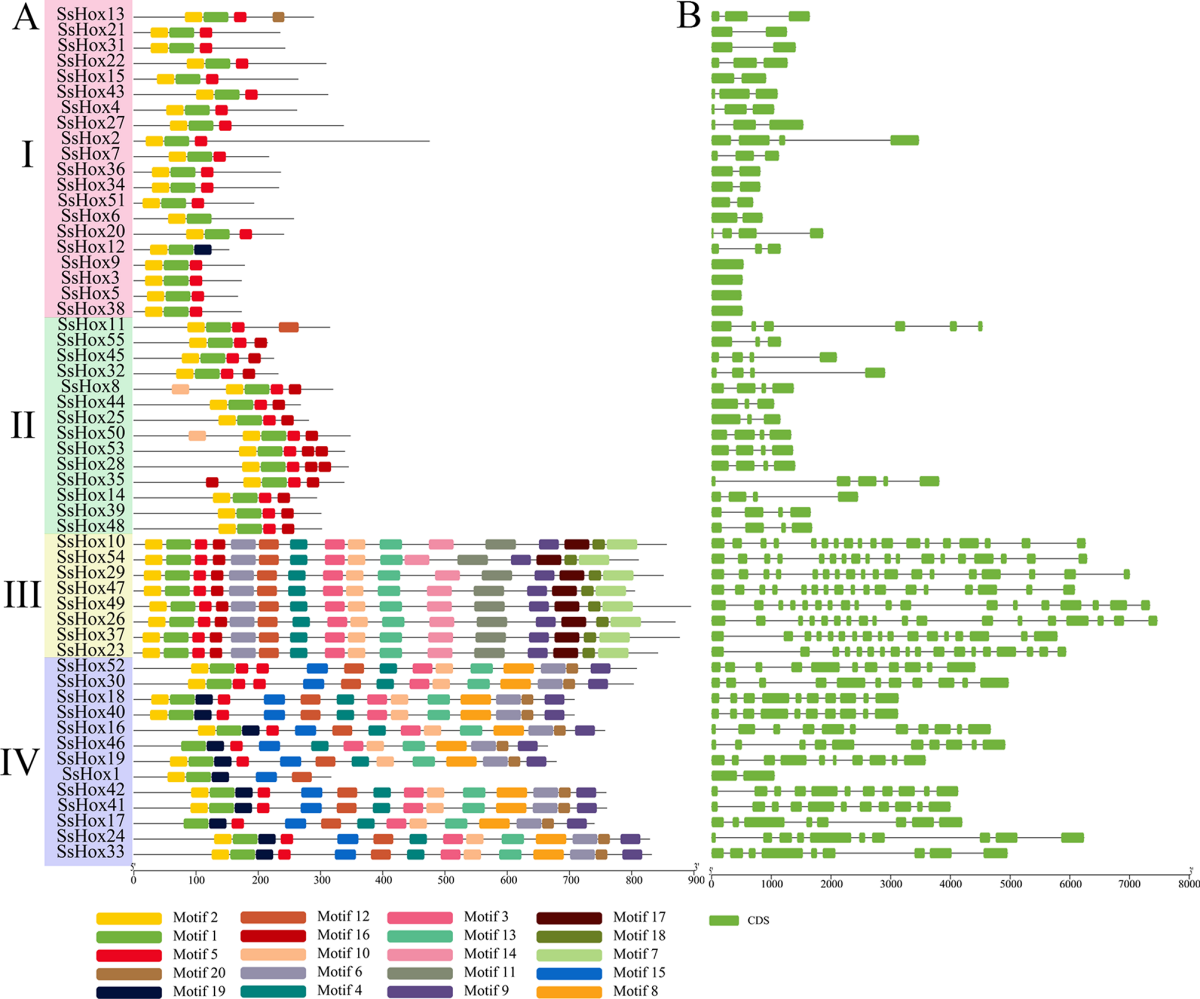
Gene structure and Conserved motifs of *HD-Zip* genes in willow. (**A**) Conserved motifs of *HD-Zip* genes in *S. suchowensis*. Distribution of the 20 conserved motifs in the *SsHD-Zip* genes following analysis by MEME tool. The different-colored boxes represent different motifs and their position in each protein sequence of *SsHD-Zip*. (**B**) Gene structure of HD-Zip genes in *S. suchowensis*. Exons are indicated by green rectangles. Gray lines connecting two exons represent introns

Motif Elicitation tool was used to predict the conserved motif of the SsHox protein, and 20 different motifs were identified. Each motif sequence identified from the MEME was annotated using the Pfam and Smart websites, and it was found that Motif1 and Motif2 encoded the HD domain, Motif3, Motif4 and Motif6 encoded the START domain, Motif5 encoded the Zip domain, and Motif7 encoded the MEKHLA domain (Table S2). Figure 3A showed that Motif1 and Motif2 were common to all members of the willow HD-Zip family, while *HD-Zip* genes of the same subfamily contained similar number and type of motifs. Moreover, HD-Zip I and HD-Zip II subfamily members have similar domains and contain fewer simple motifs, while HD-Zip III and HD-Zip IV subfamily members had more motifs and more complex domain. Domain pattern analysis also revealed that HD domain was highly conserved in all SsHox proteins (Fig. S3). Subfamily I and II possessed HD and LZ domains, while certain members of subfamily II also contained the N-terminal domain. The START domain were present in *HD-Zip* genes of the subfamilies III and IV, whereas the MEKHLA domain was specific to subfamily III.

Exon/intron structure analysis was performed on the *HD-Zip* gene of *S. suchowensis* to understand its structural diversity (Fig. 3B). The number of introns of the 55 *SsHox* genes ranged from 1 to 18, with no significant differences in the number of introns in the same subfamilies with similar structures. The subfamilies I and II had a simple gene structure with a small number of introns (1–6), while the *SsHoxs* in subfamily III contained 17 or 18. In addition, subfamily IV genes ranged in intron number from 2 to 11, with the majority of genes having 10 or 11 introns.

### Gene replication and collinearity analysis of *HD-Zip* gene family

**Fig. 4:**
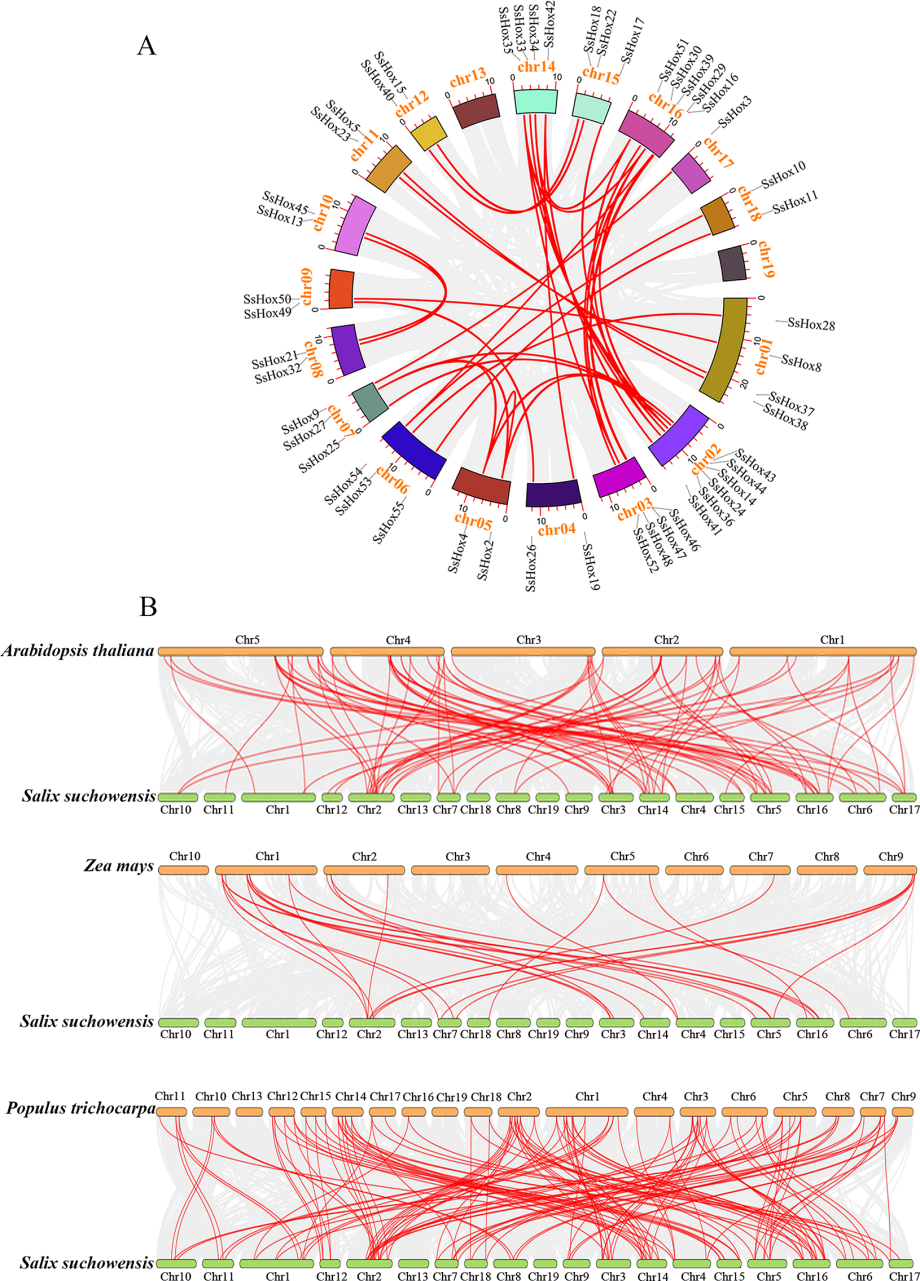
Collinearity analysis. (**A**) Collinearity analysis of *HD-Zip* gene in *S. suchowensis*. (**B**) *HD-Zip* gene collinearity between willow and other species genomes

To explore the evolutionary mechanism of the *HD-Zip* gene family in S. suchowensis, replication events in the willow genome were analyzed. A total of 36 pairs of homologous genes were obtained by the MCScanX method (Fig. 4A). These results suggested that segmental duplication might play a key role in the amplification of the *SsHD-Zip* gene family. Meanwhile, collinearity analysis was performed on the willow and three other plants (Fig. 4B). The results showed that there were 83 collinearity pairs of *HD-Zip* genes were identifed in Arabidopsis and willow, and the *HD-Zip* genes involved accounted for more than 72% of each genome. Moreover, collinearity analyses revealed that about 135 ccollinearity pairs were found between willow and poplar, and only *SsHox1* and *SsHox12* were not in the collinear regions.In addition, only 27 collinearity pairs were detected in the willow and maize, and none of the HD-Zip III members in willow were in the collinear regions. According to the results, poplar and willow had a closer genetic relationship and dicotyledons plants had a higher homology of the *HD-Zip* gene.

In order to analyze the influence of selective pressure on the evolution of the *HD-Zip* gene family, we analyzed the Ka/Ks ratio of paralogs and orthologs in four species (Table S3). The Ka/Ks ratio for all paralogues ranged from 0.07 to 0.53, while the Ka/Ks ratio of all orthologues was less than 1, indicating that the *HD-Zip* genes in willow had undergone purifying selection pressure and and had high conservation.

### GO annotation and promoter element analysis

**Fig. 5:**
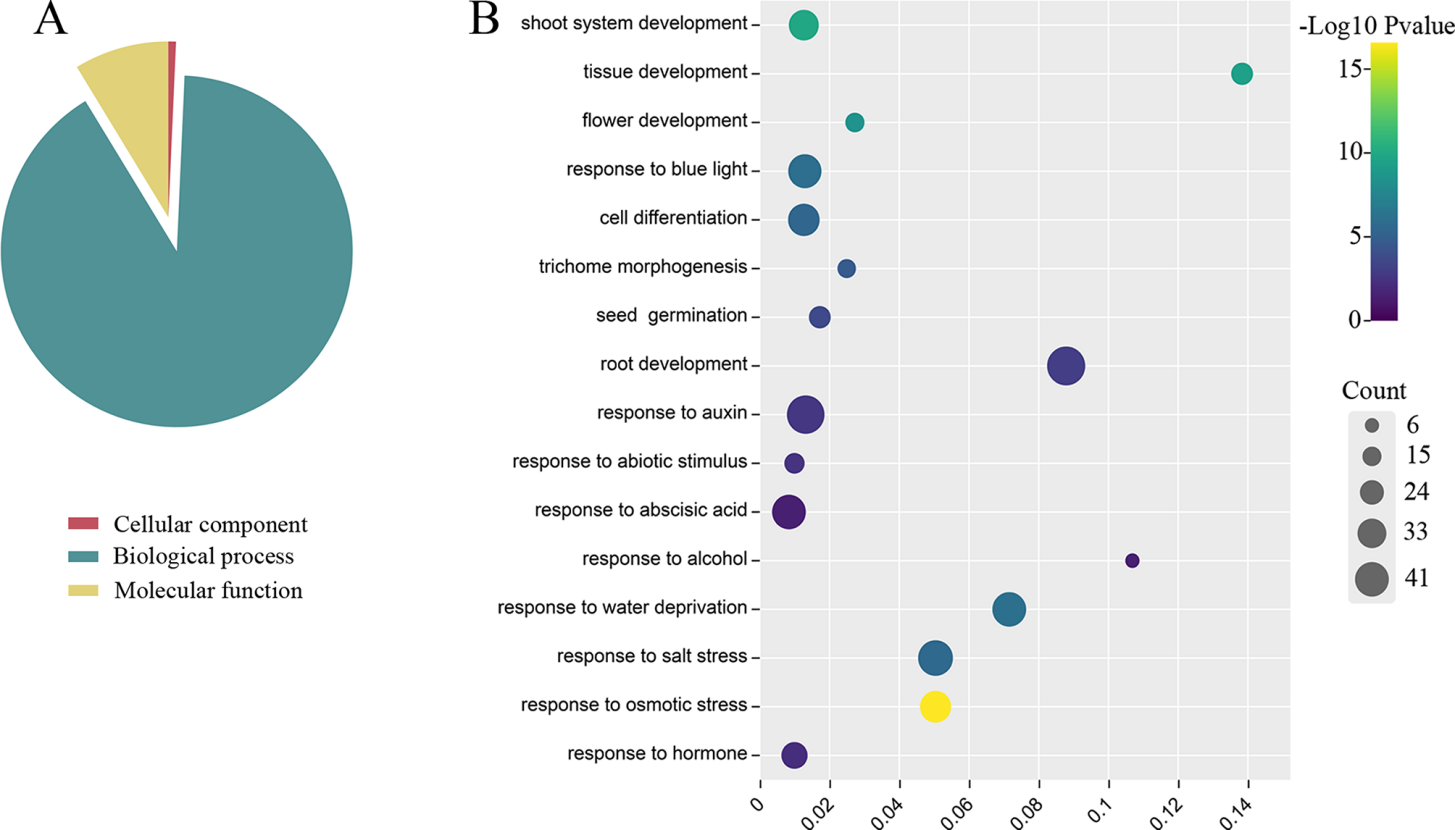
GO enrichment analysis of 55 *HD-Zip* genes in willow. (**A**) the GO annotation using a cut-off value of *P* ≤ 0.05 showed that a total of 149 GO items, including molecular function, biological process, and cellular component. (**B**) Some *SsHD-Zip* genes assigned to the categories associated with development, hormone, and stress response. The color gradient represents the size of the Pvalue and the size of circular represents number of *SsHD-Zip* genes. The X-axis shows the ratio of the number of the *SsHD-Zip* genes to the total gene number in certain categories

The GO annotation using a cut-off value of *P* < 0.05 showed that a total of 149 GO items were enriched (Table S4). The results were divided into three categories: molecular function, biological process, and cellular component. In Fig. 5A, more than 90% of the terms were categorized into biological process. Analysis of the cell component annotation revealed that HD-Zip proteins were mainly located in the nucleus, which was consistent with the prediction of subcellular localization. It was also observed that some *HD-Zip* genes were assigned to categories associated with development, hormones, and stress response (Fig. 5B). For example, 28 genes were classified in the “response to hormone” category, while 37, 42 and 44 genes were classified in the “response to osmotic stress”, “response to water deprivation” and “response to salt stress” categories, respectively.

**Fig. 6:**
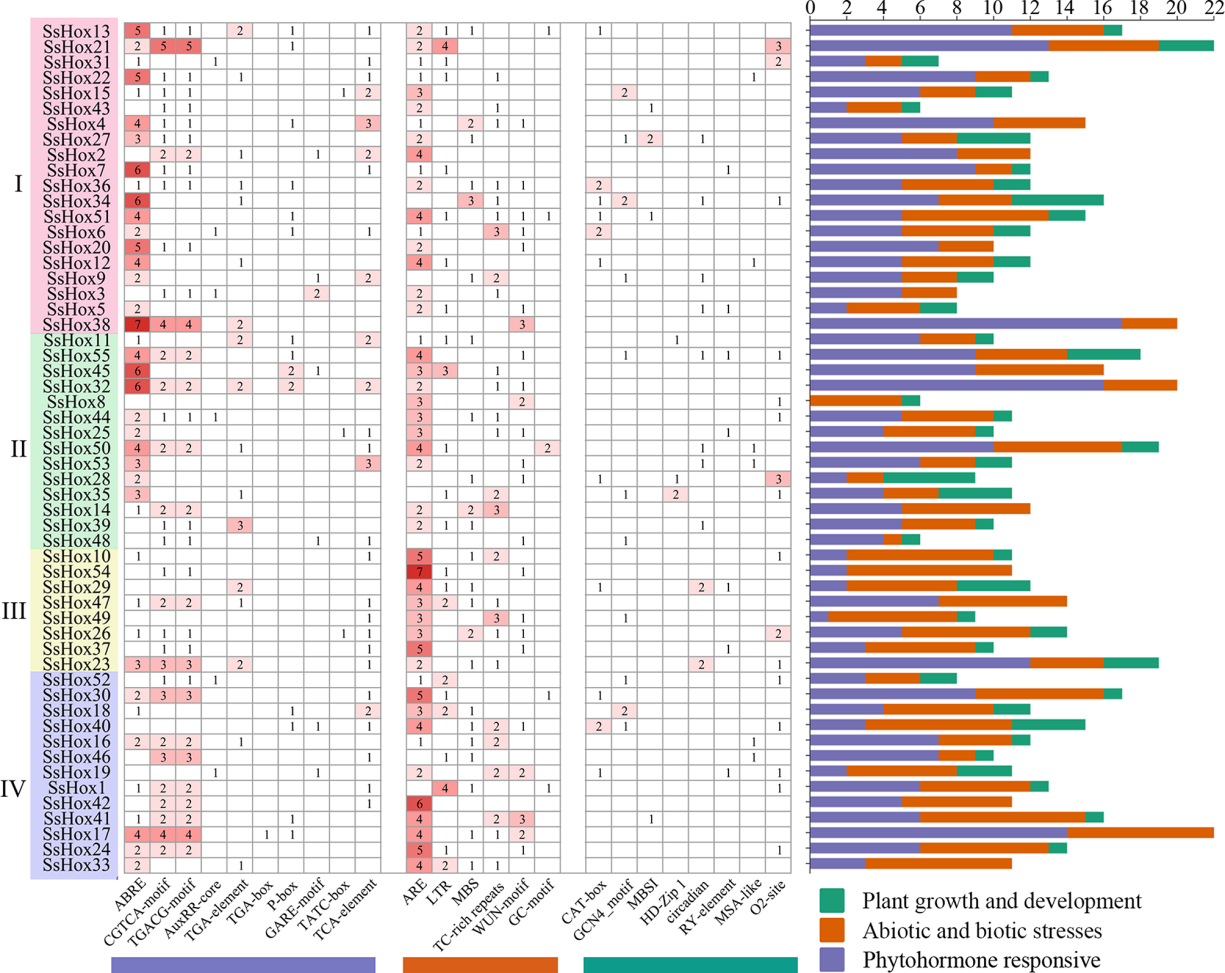
Cis-acting elements analysis of *HD-Zip* genes in promoter region of willow. Number of each cis-acting element in the promoter region (2000 bp) of *SsHD-Zip* genes

Promoter cis-acting elements are functional elements that regulate gene expression. Figure 6 showed the categorization of cis-acting elements based on their functions is illustrated, which include hormones, various stresses, and plant growth and development response elements. Many cis-elements of hormones had been found, focusing on 10 hormone response components. Of these, more than 80% and 70% of *HD-Zip* genes contained ABA-responsive elements and MeJA-responsive elements, respectively. Moreover, 274 cis-elements related to abiotic and biotic stresses were identified, including anaerobic induction response element (49.6%), low temperature response element (12.8%), wound-responsive element (10.9%), drought induced response element (10.2%), and defense stress response (14.2%). In addition, 72 elements related to plant growth and development were identified in the promoter region of the *HD-Zip* gene in *S. suchowensis*, including CAT-box, GCN4-motif, O_2_ site, HD-Zip 1, circadian, MSA-like, RY-element and MBSI element.

### Expression pattern of the *HD-Zip I* genes in *S. suchowensis* following various stresses

**Fig. 7:**
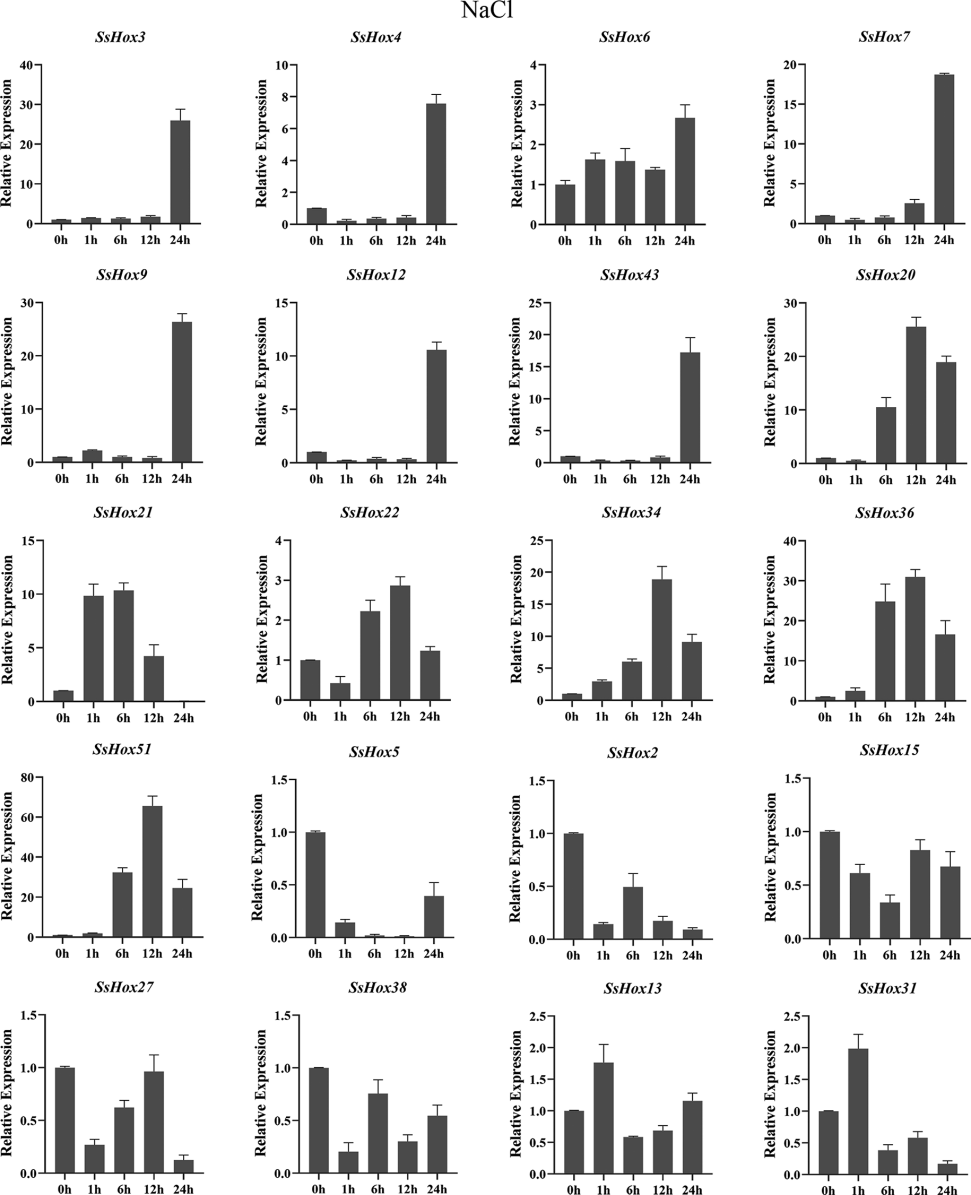
Expression analysis of *HD-Zip I* genes following NaCl treatments by qRT-PCR. The Y-axis and X-axis indicates relative expression levels and the time courses of stress treatments, respectively. Mean values and standard deviations (SDs) were obtained from three biological and three technical replicates. The error bars indicate standard deviation

**Fig. 8:**
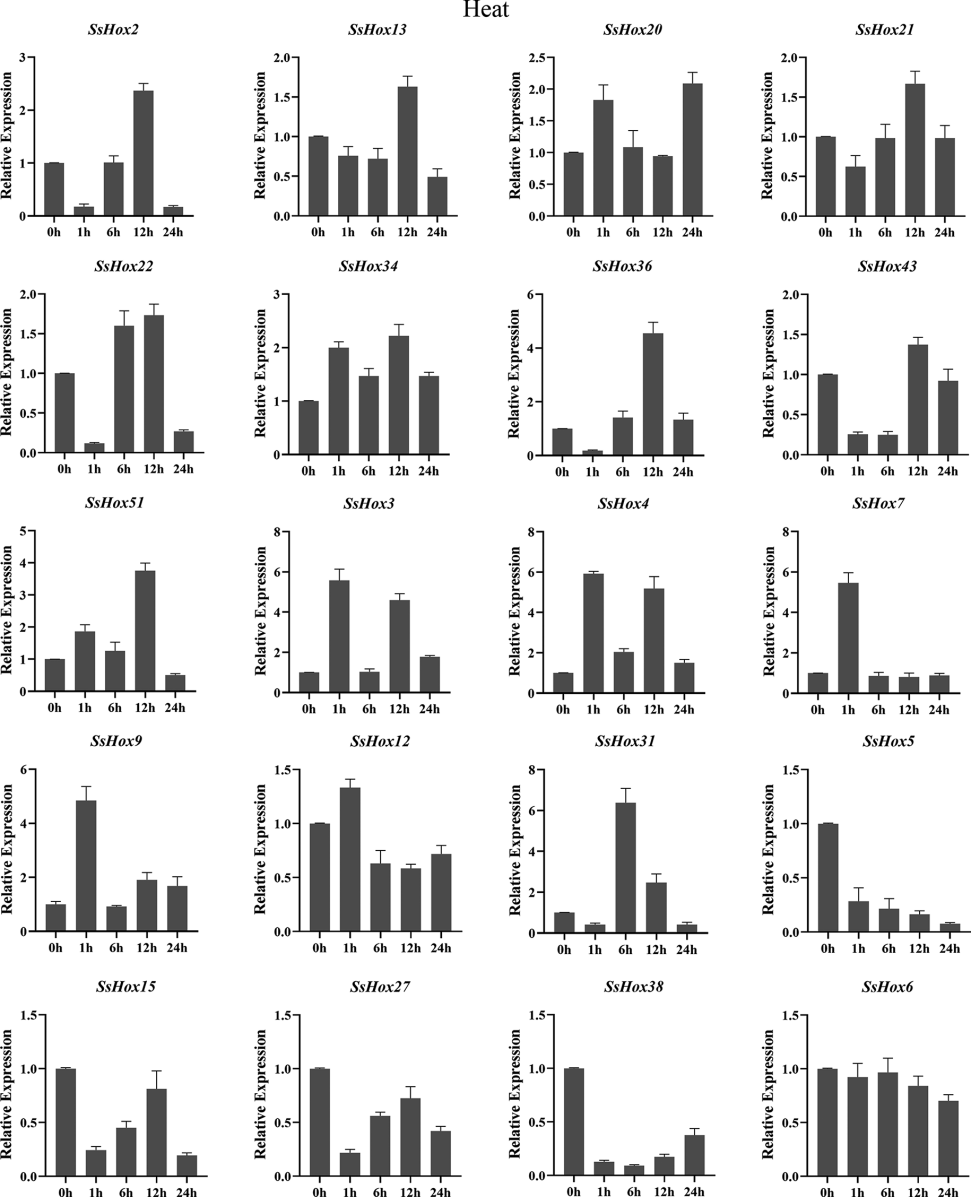
Expression analysis of *HD-Zip I* genes following heat treatments by qRT-PCR. The Y-axis and X-axis indicates relative expression levels and the time courses of stress treatments, respectively. Mean values and standard deviations (SDs) were obtained from three biological and three technical replicates. The error bars indicate standard deviation

**Fig. 9:**
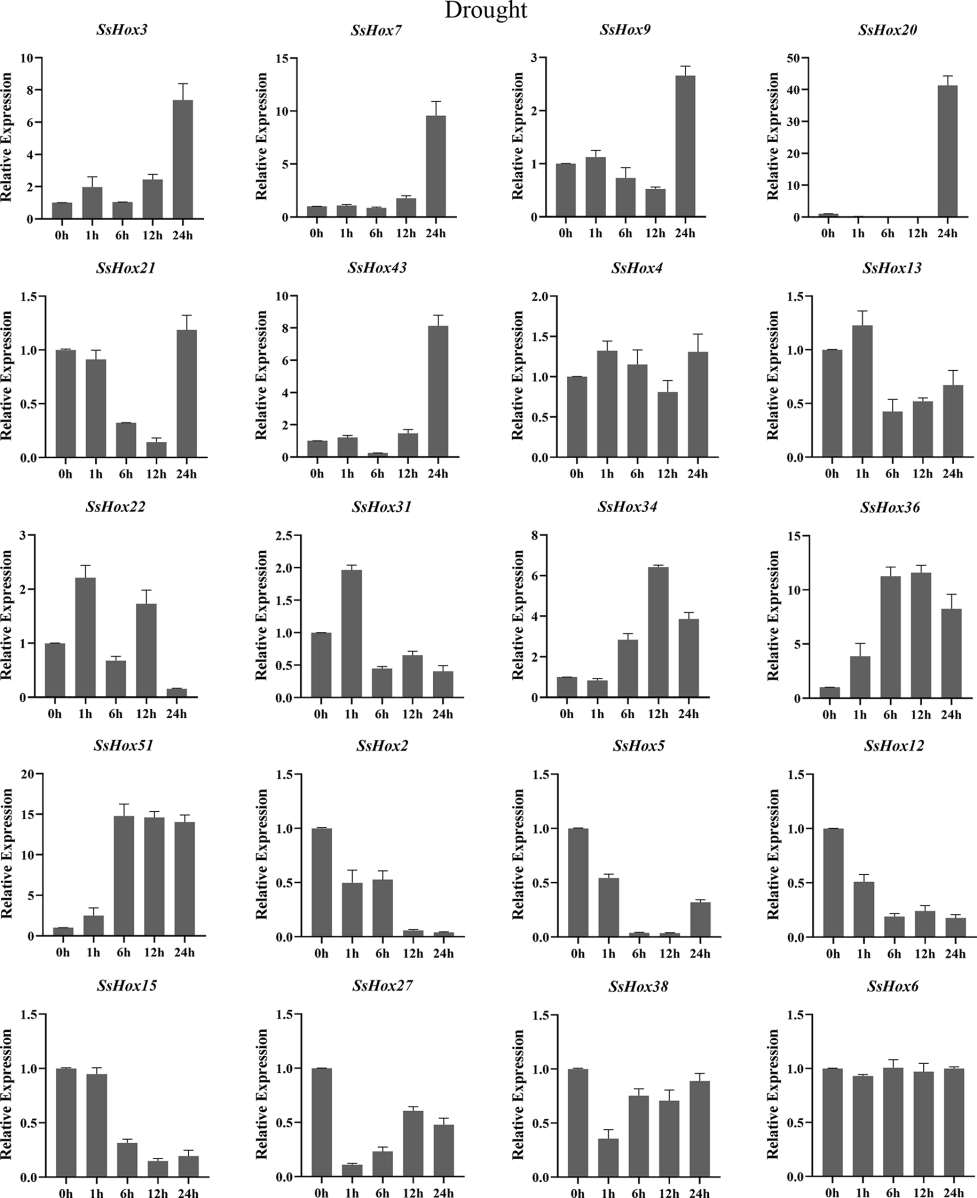
Expression analysis of *HD-Zip I* genes following drought treatments by qRT-PCR. The Y-axis and X-axis indicates relative expression levels and the time courses of stress treatments, respectively. Mean values and standard deviations (SDs) were obtained from three biological and three technical replicates. The error bars indicate standard deviation

The *HD-Zip I* genes has been reported to play an important role in the plant’s response to abiotic stres [30, 31]. Therefore, we investigated the expression patterns of willow *HD-Zip I* genes under PEG, heat and NaCl treatments. During the NaCl stress, the seven genes were significantly upregulated at 24 h of treatment, including *SsHox3*, *4*, *6*, *7*, *9*, *12* and *43* (Fig. 7). However, five SsHoxs were down-regulated apparently. Additionally, *SsHox34/-36*, *SsHox34/-51* and *SsHox51/-36* showed the same expression pattern after NaCl treatment. For example, the expression of *SsHox34* was up-regulated and reached a maximum at 12 h, but then gradually decreased. As for the heat stress (Fig. 8), *SsHox5*, *SsHox15*, *SsHox27*, and *SsHox38* were downregulated by heat treatment across all time points. The expression level of 5 *SsHox* genes exhibited a rapid rapid strong up-regulation and peaked at 12 h after exposure to high temperature, but 9 *SsHox* genes were significantly up-regulated at 1 h. Interestingly, the expression of *SsHox31* differed from other *SsHox* genes, being slightly down-regulated at the first time point, drastically up-regulated sixfold at 6 h, but then gradually decreased at subsequent time points. Additionally, qRT-PCR was performed to investigate the response to PEG treatment (Fig. 9). Of the 20 *HD-Zip* genes, 19 were up-regulated or down-regulated at some time points, while only *SsHox6* was not expressed at all time points. Notably, *SsHox20* was up-regulated more than 40-fold of drought stress. Furthermore, five paralogs (*SsHox2/-27*, *SsHox3/-9*, *SsHox34/-36*, *SsHox34/-51* and *SsHox51/-36*) exhibited similar expression patterns in response to PEG treatment. For example, the expression level of *SsHox3/-9* was significantly up-regulated and peaked at 24 h. However, the expression patterns of two paralogous genes (*SsHox2/-43* and *SsHox27/-43*) were opposite. Like *SsHox2/-43*, *SsHox2* was continuously down-regulated, whereas *SsHox43* remained up-regulated at its maximum value.

### Correlations and coregulatory networks of *SsHD-Zip I* genes under various stresses

**Fig. 10:**
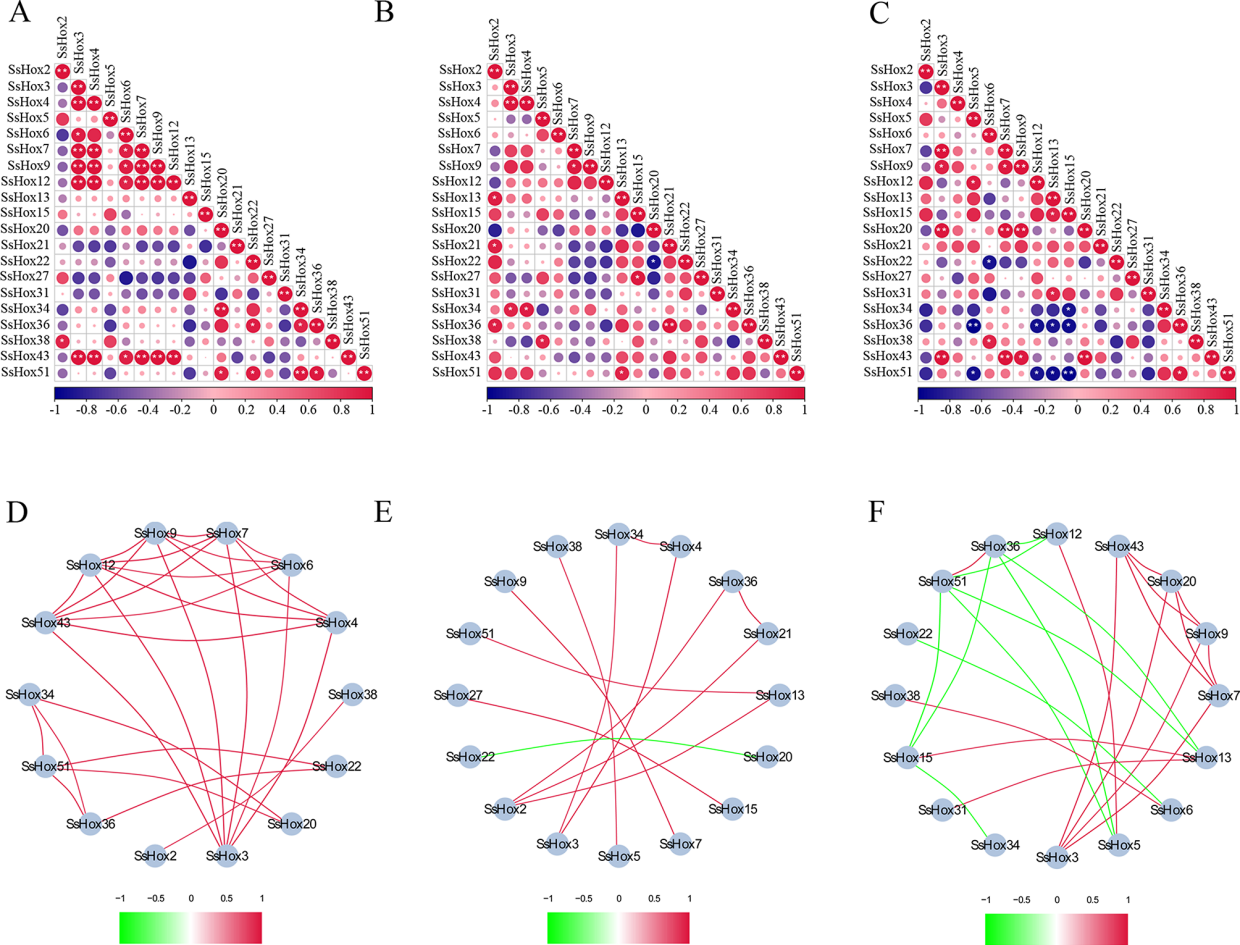
Correlations among *HD-Zip I* genes under NaCl, PEG and heat treatment. Correlation analysis of *HD-Zip I* genes under NaCl (**A**), heat (**B**) and PEG (**C**) treatment was performed based on the PCCs of qRT-PCR data. Correlations are indicated by the size and colour of circles. The lower bar represents the PCC values. * and ** represent correlations with P-value ≤ 0.05 and P-value ≤ 0.01, respectively. The coregulatory network of *HD-Zip I* genes under NaCl (**D**), heat (**E**) and PEG (**F**) treatment was illustrated by Cytoscape. The significant PCCs of gene pairs (P-value ≤ 0.05) are included, and the different correlation levels of the gene pairs are marked by edge lines with different colors, as shown below the coregulatory networks

Based on the PCCs of their relative expression levels of *HD-Zip I* genes, correlation and coregulatory networks were constructed to examine the connections between genes in response to PEG, NaCl, and heat treatment. Under salt treatment, positive correlations and coregulatory network were observed between *HD-Zip I* genes. Under salt treatment, positive correlations (Pvalue ≤ 0.05 and 0.8 < PCC) were observed between *SsHoxs*, such as *SsHox51*, *SsHox36*, and *SsHox22* (Fig. 10A and D). Among them, *SsHox3*, *SsHox9*, *SsHox7*, *SsHox20*, and *SsHox43* also showed positive correlations with each other under the PEG treatments (Fig. 10C and F). Moreover, 9 gene pairs showed negative correlations (Pvalue ≤ 0.05 and − 1.0 < PCC< -0.8) in response to the PEG treatments. In addition, there was a significant positive correlation between 14 *SsHoxs* under heat stress (Fig. 10B and E), and only *SsHox22*/*SsHox20* showed a negative correlation.

## Discussion

Plant transcription factors often interact with DNA and other proteins or transcription factors to promote or inhibit gene expression, thus participating in plant growth and development and response to stress [32]. With the development of molecular bioinformatics, the *HD-Zip* gene family has been identified in more and more plants. In this study, we used bioinformatics and qRT-PCR analyses to perform genome-wide analysis of willow *HD-Zip* genes to investigate their regulatory roles in stress response.

A total of 55 *HD-Zip* genes were identified in willow, distributed unevenly over 18 chromosomes (Figs. 1 and 2). There are 15 members of the HD-Zip I subfamily in Arabidopsis, 21 in poplar and 17 in maize [33, 34]. The genome sizes vary greatly between species, but the number of genes in the same subfamily is similar, indicating that the evolution of genes in this subfamily is relatively conservative. Previous studies have shown that the *HD-Zip I* genes in Arabidopsis and maize can be further subdivided into eight subclasses (α, β1, β2, γ, δ, ε, ζ and φ) (Fig. S2), and these subclasses may have a common origin in early organisms [35]. However, subclasses β1 and ζ did not contain *HD-Zip I* genes in poplar and willow. One of the ζ subclass contained only the maize *HD-Zip I* genes, suggesting that maize acquired an additional branch. According to the structural similarity, evolutionary relationship analysis and motif distribution of the *HD-Zip* gene family, the willow *HD-Zip* gene family could be divided into 4 subfamilies. Among these 4 subfamilies, the HD-Zip III subfamily had the least number of members. This was consistent with the findings in poplar, watermelon and *Zanthoxylum armatum* [33, 36, 37]. It was found that members of different subfamilies differ greatly in sequence length, exon number and conserved domain (Fig. 3). For example, *SsHox37* (HD-Zip III) had 17 exons and the longest protein sequence, whereas *SsHox12* (HD-Zip I) had 3 exons and the shortest protein sequence. In addition, the gene structure and conserved motifs of most genes in the same subfamily are similar, which may be related to the function and phylogenetic clustering of the family.

Collinearity analysis revealed that segmental duplications containing *SsHoxs* could be detected on all chromosomes except chromosome 13 and 19, indicating that segmental duplications were the main cause of the expansion of the *SsHD-Zip* gene family (Fig. 4). Similar results of collinearity analysis had been found in other species such as potato and peach [26, 38]. The collinear pairs of HD-Zip family genes between willow and poplar genomes were more than those between other genomes, indicating that willow and poplar were closely related. Moreover, genes with collinear relationships could also be grouped into the same class in the phylogenetic tree, indicating that these genes are relatively conserved in the process of genome evolution. These results may also be related to the conserved domains of these genes. Combined with the promoter analysis and GO annotation (Figs. 5 and 6), it was found that there were many cis-acting elements related to growth and development, stress response and hormone response in the promoters of many *SsHD-Zip* genes, and most of the genes were annotated to the enriched categories related to salt, osmotic stress and water deprivation response. It is speculated that the willow HD-Zip family may be transcriptionally regulated in adverse growth environments.

Many HD-Zip I subfamily genes have been reported to be involved in the regulation of abiotic stresses such as drought, salinity and temperature stress [6, 39]. For example, the *HaHB4* gene of sunflower HD-Zip I subfamily regulated drought resistance through ethylene-mediated senescence, and the *PeHDZ* genes of Moso bamboo HD-Zip I subfamily were significantly induced by PEG and NaCl [40, 41]. In contrast to subfamilies II, III, and IV, the HD-Zip I subfamily plays an important role in response to abiotic stresses [1]. Therefore, we investigated the expression patterns of willow *HD-Zip I* genes in response to PEG, salt and heat treatment by qRT-PCR analysis, and the expression of most genes increased under stress (Figs. 7, 8 and 9). The expression levels of *SsHox5*, *SsHox28* and *SsHox23* were suppressed under all three stresses. On the contrary, *SsHox3*, *SsHox7*, *SsHox9*, *SsHox36* and *SsHox51* were strongly expressed under the different treatments. These five genes may play an important role in the response of willow to abiotic stress. *SsHox36* and *SsHox51* were homologous to *AtHB7* and *AtHB12*, while two Arabidopsis genes were induced by ABA, drought, and salt stress, and improve plant drought resistance by influencing stomatal closure [42]. Moreover, *SsHox36* and *SsHox51* were homologous to *PsnHDZ63* (Potri.002G176300.1), and overexpression of *PsnHDZ63* confers salt tolerance in transgenic plants [43]. These results suggested that *SsHox36* and *SsHox51* may play essential roles in responses to PEG, heat and NaCl treatment. At the same time, some genes were discovered to be paralogous pairs whose expression levels differed significantly under a given stress treatment, in particular the expression patterns of *SsHox3/-9*, *SsHox34/-36*, *SsHox34/-51* and *SsHox51/-36* differed under all three stresses. It is possible that some paralogous pairs have functionally diverged during the evolutionary process. Under 37 °C high temperature treatment, the expression levels of *SsHox36*, *SsHox3*, *SsHox4*, *SsHox7*, *SsHox9* and *SsHox31* were strongly up-regulated (more than 4-fold) at some points. Similarly, 6 HD-Zip I genes of potato were differentially expressed in different tissues at high temperature (37 °C) [38]. Other *HD-Zip* genes such as *HaHB1*, *AtHB13* and *TaHDZIPI-5* had been implicated d in plant cold tolerance [44, 45].

## Conclusion

In this study, 55 *HD-Zip* genes of *S. suchowensis* were identified, which were unevenly distributed on 18 out of 19 chromosomes. The willow HD-Zip genes were classified into four subfamilies using phylogenetic analysis and conserved domain analysis. Results from GO annotation and promoter analysis showed that the *SsHox* gene was controlled by a complex regulatory network. Furthermore, combining with the results of *HD-Zip I* gene expression analysis in willow, it was speculated that *HD-Zip I* gene played an important role in the resistance to abiotic stress, but the specific functions of each gene needed to be further studied. In this study, the whole genome of the willow *HD-Zip* gene family members was identified and analyzed, providing a theoretical basis for the subsequent functional verification of this gene family.

## Materials and methods

### Identification of putative *HD-Zip* genes in *Salix suchowensis*

The protein sequence of *S.suchowensis* were downloaded from the website (https://figshare.com/articles/dataset/Willow_gene_family/9878582/1?file=17720912, accessed 20 December 2022). The Hidden Markov model of the *HD-Zip* gene family domain (PF00046) from the Pfam database (http://pfam.xfam.org/, accessed 22 December 2022) were used to search the HD-Zip genes of *S.suchowensis.* And a local blast search of the *S.suchowensis* protein database was performed using the BlastP tool (E value-5) in TBtools, with 63 protein sequences of poplar HD-Zip as query sequences. After manual de-duplication, the 64 putative *HD-Zip* genes in *S.suchowensis* were obtained. Meanwhile, the NCBI Conserved Domain Database and the SMART database were used to verify 64 putative genes, leaving candidate genes that included the known conserved domains (HD domain). ExPASy (http://www.expasy.ch/tools/pi_tool.html, accessed 26 December 2022) and WoLP PSORT (https://wolfpsort.hgc.jp/, accessed 30 December 2022) were used to determine the molecular weight, isoelectric point and localization for each *HD-Zip* genes from *S.suchowensis*.

### Multiple alignment and phylogenetic analysis

Multiple sequence alignments of the full-length HD-Zip protein sequences from Populus, Arabidopsis, maize and *S.suchowensis* were performed using ClustalW in MEGA 11.0.10 with default parameters. With default settings and a bootstrap value of 1000, we created a phylogenetic tree using the neighbor-joining method (NJ) and Maximum Likelihood (ML) in the MEGA 11.0.10 program [46].

### Chromosomal distribution, collinearity and Ka/Ks analysis

The chromosomal locations of the *HD-Zip* genes in *S.suchowensis* were extracted from the GFF3 annotation file downloaded from the website (https://figshare.com/articles/dataset/Willow_gene_family/9878582/1?file=17720912, accessed 20 December 2022) and were displayed using TBtools-II v2.003 software [47]. Genome annotation file of Arabidopsis, maize and poplar were obtained from Phytozome database. One Step MCScanX-Super Fast in TBtools-II v2.003 was used to analyze the genome-wide collinearity between willow and three other species, and the collinear results were mapped using TBtools-II v2.003 with default settings [48]. The ratio of non-synonymous to synonymous substitutions (Ka/Ks) of orthologues and paralogues was calculated by TBtools-II v2.003.

### Prediction of gene structure, conserved motifs, and cis-regulatory elements

The exon and intron location information of *SsHD-Zip* genes were extracted from the GFF3 annotation file, and the results were uploaded to the Gene Structure Display Server 2.0 website. To predict and analyze conserved protein motifs, all candidate SsHD-Zip protein sequences were uploaded to the MEME online tool [49]. A maximum of 20 motifs were set, while all other parameters were kept as default. The 2 kb sequences upstream of each *SsHD-Zip* gene were retrieved from the Genome annotation file. These sequences were then submitted to PlantCARE (http://bioinformatics.psb.ugent.be/webtools/plantcare/html/) for identification and prediction of Cis-elements.

### Gene ontology annotation analysis

Gene ontology (GO) analysis was conducted for the SsHox genes using the agriGO database (http://systemsbiology.cau.edu.cn/agriGOv2/index.php, accessed 15 May 2023). The reference set consisted of all 26,599 genes of *S.suchowensis*, while the test set included 55 *SsHox* genes. The GO analysis diagram was generated using ChiPlot (chiplot.online).

### Plant materials, growth conditions, and stress treatments

*S. suchowensis* were cultured by hydroponics (Hoagland’s nutrient solution) for six weeks in plant climate incubator (16 h light/8 h dark and 25 /22°C day/night). In order to investigate the expression profiles of *SsHD-Zip* I genes under abiotic stresses, the seedlings were treated under 200 mM NaCl, 20% (w/v) polyethylene glycol (PEG 6000), 37 °C, respectively. All leaves were harvested at 0, 1, 6, 12 and 24 h after each treatment, and then rapidly frozen in liquid nitrogen and stored at -80 °C for total RNA extraction.

### RNA extraction and qRT-PCR analysis

Total RNA was extracted from the samples using the Aidlab plant RNA kit (Aidlab Biotech, Beijing, China) and the first-strand cDNA was synthesized using the UnionScript First-strand cDNA Synthesis Mix (Gensand, Bejing, China). The OTU (OTU-like cysteineprotease familyprotein) gene was used as the reference gene for salt stress, and UBC (Ubiquitin-conjugating enzyme E2) both for heat and drought treatment [50, 51]. All primers for qRT-PCR experiments were designed with Primer 5.0 and checked for primer specificity with TBtools (Table S5). Real-time PCR was performed on a CFX96TM Real-Time System (BIO-RAD, CA, USA) with TB Green Premix Ex Taq II (Tli RNaseH Plus; TaKaRa Biotechnology) in a sample volume of 10 µL. For each sample, we conducted three biological and three technical replicates. In addition, the relative expression level of each gene was calculated by standard 2^−∆∆CT^ method was calculated [52].

### Statistical and Pearson correlation analysis

The mean values and standard deviations (SDs) were calculated from three biological and three technical replicates. Pearson correlation coefficients (PCCs) and p-values of stress-induced *SsHox* gene pairs were obtained from the qRT-PCR results and plotted using the R package. The coexpression network was constructed in Cytoscape v3.9.1 [53] by including gene pairings with PCC values greater than 0.8 and significant at the 0.05 significance level (P-value). Significant differences are indicated at **P < 0.01 and *P < 0.05.

### Electronic supplementary material

Below is the link to the electronic supplementary material.

Supplementary Tables

Supplementary Figures

## Data Availability

The genome sequences of *Salix suchowensis* were downloaded from the website (https://figshare.com/articles/dataset/Willow_gene_family/9878582/1?file=17720912, accessed 20 December 2022). The genome sequences of *A. thaliana* were downloaded from Phytozome database (https://phytozome-next.jgi.doe.gov/info/Athaliana_TAIR10, accessed 22 December 2022). The genome sequences of rice were downloaded from Phytozome database (https://phytozome-next.jgi.doe.gov/info/Osativa_v7_0, accessed 22 December 2022). The genome sequences of maize were downloaded from Phytozome database (https://phytozome-next.jgi.doe.gov/info/Zmays_RefGen_V4, accessed 22 December 2022). The genome sequences of poplar were downloaded from Phytozome database https://phytozome-next.jgi.doe.gov/info/Ptrichocarpa_v4_1, accessed 20 December 2022). The datasets supporting the results of this article are included in the article and Additional files.
