# Supplementary material for: Comprehensive bioinformation analysis of homeodomain-leucine zipper gene family and expression pattern of HD-Zip I under abiotic stress in Salix suchowensis
Source: BMC Genomics. 2024 Feb 15;25:182. doi: 10.1186/s12864-024-10067-x (PMC10870566; doi:10.1186/s12864-024-10067-x)
Supplement: Supplementary file 2 — Supplementary Figures [file 12864_2024_10067_MOESM2_ESM.docx]

**Supplementary Figures**


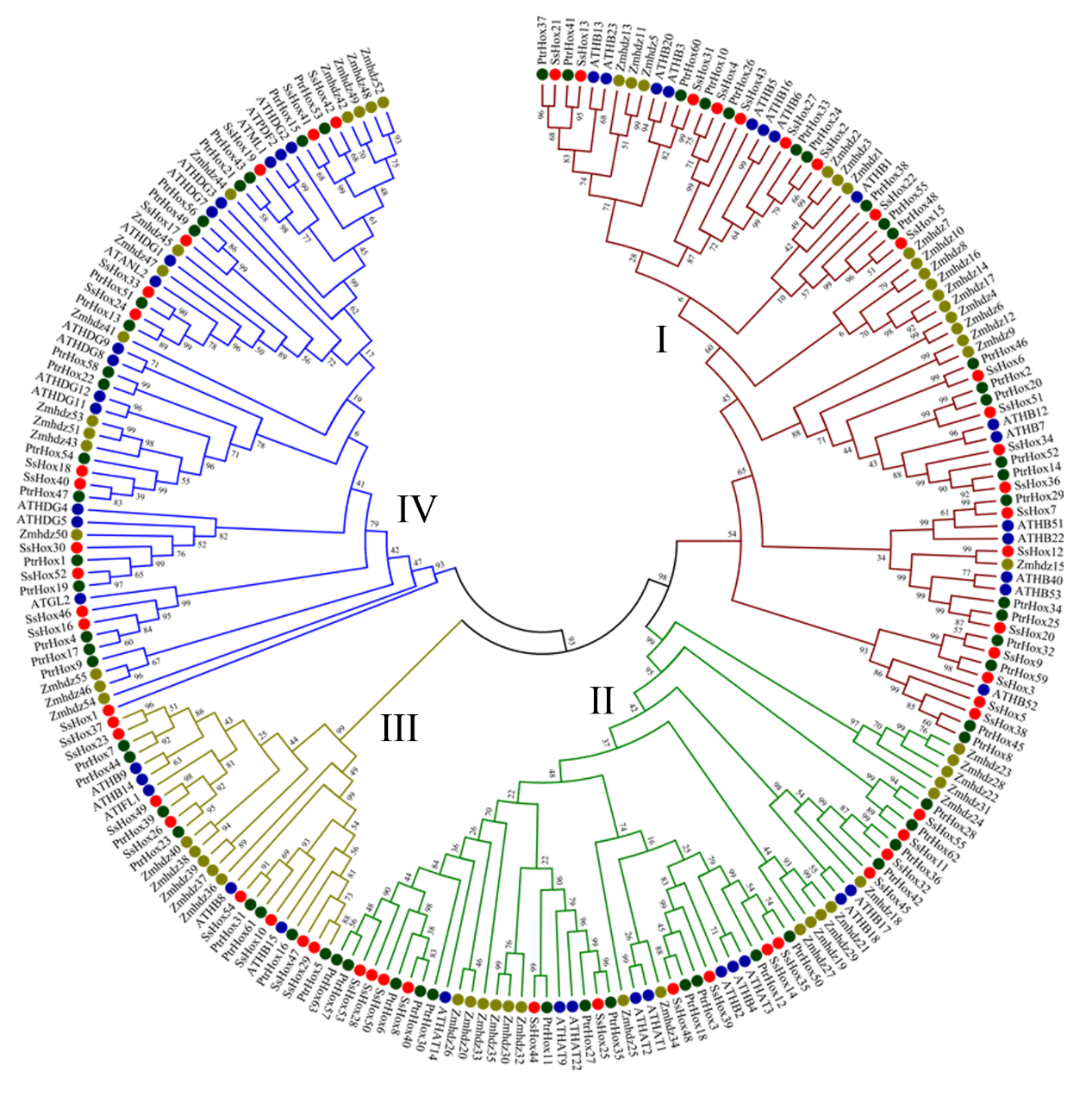


**Fig. S1 Phylogenetic tree of *HD-Zip* genes from willow, Arabidopsis, poplar and maize by Maximum** **Likelihood method.**

Classification of *HD-Zip* gene family based on phylogenetic tree. 55 *SsHD-Zip* genes, 44 *AtHD-Zip* genes, 63 *PtHD-Zip* genes and 55 *ZmHD-Zip* genes are clustered into four subfamilies (I-IV). *HD-Zip* genes from *S. suchowensis*, Arabidopsis, poplar and maize are denoted by red, blue, green and yellow shape, respectively.


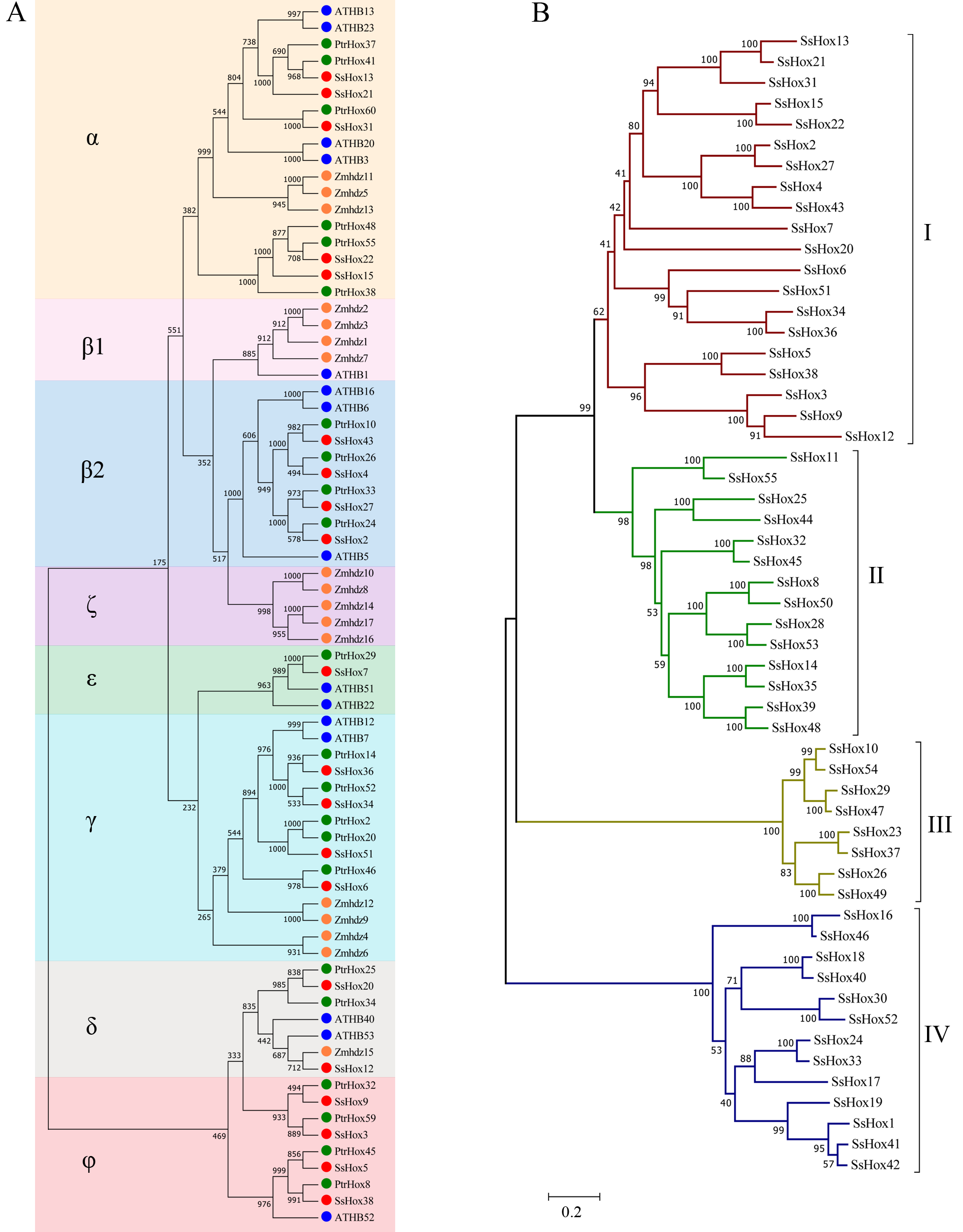


**Fig. S2 Phylogenetic tree of *HD-Zip* genes.**

(A) Phylogenetic tree based on full-length HD-Zip I protein sequences from *S. suchowensis*, poplar, maize, and Arabidopsis. (B) Phylogenetic tree of *HD-Zip* genes from *S. suchowensis*.


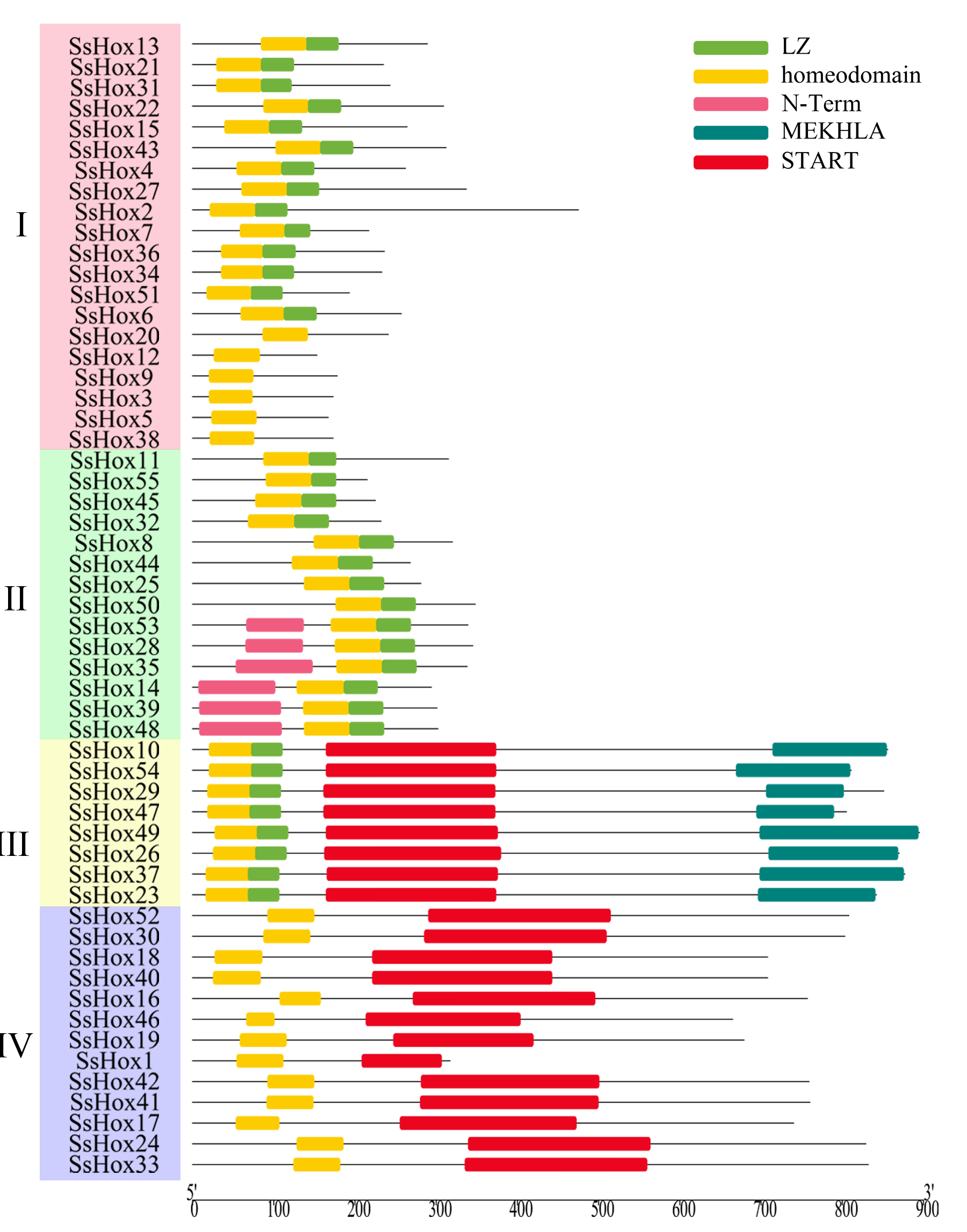


**Fig. S3 Domain pattern of HD-Zip genes from *S. suchowensis*.**
